# A New Score for Metabolic Age in Type 2 Diabetes Mellitus: Physical Rating Score

**DOI:** 10.3390/jcm14092868

**Published:** 2025-04-22

**Authors:** Hasan Esat Yücel, Tufan Ulcay, Özkan Görgülü, Ruken Öncü, Emre Uğuz, Erkan Dulkadiroğlu

**Affiliations:** 1 Department of Internal Medicine, Faculty of Medicine, Ahi Evran University, Kırsehir 40100, Turkey; erkan_dulkadir@hotmail.com; 2 Department of Anatomy, Faculty of Medicine, Ahi Evran University, Kırsehir 40100, Turkey; tufanulcay@gmail.com (T.U.); ruken.oncu@ahievran.edu.tr (R.Ö.); emre.uguz@ahievran.edu.tr (E.U.); 3 Department of Biostatistics and Medical Information, Faculty of Medicine, Ahi Evran University, Kırsehir 40100, Turkey; ozkangorgulu@gmail.com

**Keywords:** body mass index, chronological age, metabolic age, physical rating score, type 2 diabetes mellitus, bioimpedance analysis

## Abstract

**Background**: Metabolic age (met-age), a risk marker, may vary within the same chronological age group. Its association with chronological age, waist/height ratio, obesity degree, body mass index (BMI), and physical rating score (PRS)—risk factors for type 2 diabetes—remains unexplored. **Methods**: A total of 122 type 2 diabetes patients (50 males, 72 females) were analyzed. Bioimpedance measurements were taken using the Tanita MC-780MA, alongside anthropometric data. Variables were compared, and correlations with met-age were assessed. **Results**: The met-age–chronological age difference was higher in females with type 2 diabetes (*p* < 0.001). Females also had higher BMI, obesity degree, fat mass, and waist/height ratio (*p* < 0.05), but lower waist/hip ratio (*p* < 0.001). Males showed higher PRS, muscle mass, and fat-free mass (*p* < 0.001). Met-age positively correlated with chronological age and negatively with PRS in both sexes (*p* < 0.05). According to the linear regression model, in females, the met-age was influenced positively by chronological age and waist/height ratio and negatively by PRS (R^2^ = 0.983). In males, it was positively influenced by chronological age, obesity grade, and BMI, but negatively by PRS (R^2^ = 0.974). **Conclusions**: Met-age correlates with chronological age and PRS in type 2 diabetes patients. It increases with advancing age and lower PRS, with waist/height ratio impacting females and BMI/obesity degree affecting males.

## 1. Introduction

Type 2 diabetes mellitus (T2DM) is a chronic metabolic disease characterized by hyperglycemia due to insulin secretion deficiency or resistance and impaired carbohydrate, lipid and protein metabolism [1]. Obesity, which is associated with increased body fat and insulin resistance, is an important risk factor for T2DM and increases the incidence of the disease [2]. However, in individuals with diabetes, an increase in body fat mass occurs as a result of the disease and metabolic unhealthy body compositions may develop [3]. Also, the risk of sarcopenia in patients with diabetes is three times greater than that in nondiabetic patients and is associated with an increased risk of death [4,5]. In T2DM, hyperglycemia causes an increase in fat mass and a decrease in muscle mass, and these effects can be demonstrated by bioimpedance analysis (BIA) [6]. BIA is a noninvasive, inexpensive, safe and easily applied method that measures body composition and metabolic status based on the electrophysiological properties of the human organism [7]. BMI, degree of obesity, body fat/muscle ratios and met-age can be obtained via BIA [8,9,10]. Met-age is a new risk marker that was introduced to explain the differences in the basal metabolic rate (BMR) of individuals in the same chronological age group and is easily understood by patients [11]. For example, if an individual’s met-age is older than their chronological age, the BMR decreases and the risk of cardiovascular disease (CVD) increases [12]. Furthermore, met-age serves as a predictor of metabolic syndrome; a met-age exceeding chronological age suggests a heightened risk of metabolic syndrome, while a met-age that is lower indicates favorable metabolic health [13]. The physical rating (PR) is a scale obtained by BIA and defines various body types according to the fat and muscle ratios in the body [13]. With Tanita, the PRS based on fat and muscle mass is calculated out of one hundred points on the electronic analyzer and obtained with professional software.

The body of literature investigating met-age remains limited, with existing studies suggesting its potential role as a marker of increased risk and predictive value [10,11,13]. However, to date, no studies have explored this parameter in the context of T2DM. The main aim of our study was to investigate how chronological age, PRS and anthropometric measurements affect met-age in T2DM. Additionally, we seek to investigate sex-specific variations in these associations to provide deeper insights into the underlying dynamics.

## 2. Materials and Methods

Our study was conducted prospectively in T2DM patients admitted to the Internal Medicine Clinic of Kırşehir Training and Research Hospital between October and November 2024, and a total of 122 patients (male: 50, female: 72) were included in the study. Male and female patients with T2DM older than 18 years were included in the study. The chronological age of the participants was calculated from the date of birth. Malignancy, acute or chronic infection, acute complication states of diabetes (hypoglycemia, diabetic ketoacidosis, ketotic hyperosmolar coma and lactic acidosis), acute myocardial infarction, acute cerebrovascular disease, cardiac arrhythmia, pacing, pregnancy and female patients during menstrual cycle were excluded.

The study was approved by the Ethics Committee of Kırşehir Ahi Evran University Faculty of Medicine (Ethics number: 2024-14/116, approval date 6 August 2024). All procedures were performed in accordance with the Declaration of Helsinki, and informed consent was obtained from all subjects.

### 2.1. Anthropometric and BIA Measurements

Anthropometric measurements of the participants were performed during the first examination after 8–10 h of fasting. Height was measured with an automatic stadiometer BSM 370 (Biospace Co., Seoul, Republic of Korea) with an accuracy of 0.1 cm while the subject was standing without shoes. BIA data of the participants were measured by Tanita MC-780MA (Tanita Corporation, Tokyo, Japan) with electrical current obtained at 5 kHz frequency. Patients stood on the device with the electrodes touching the soles of their feet and, at the same time, were instructed to hold the hand probes with their bare hands, with the electrodes on the hand probes touching their hands and feet, and to remain in an upright position, motionless and stationary until the results were displayed on the screen and measurements were taken. In the weight measurement, the weight of the fixed garment was recorded on the analyzer as 1 kg on average. Body weight (kg), met-age, PRS, degree of obesity, fat mass, muscle mass and lean mass measurements were obtained with Tanita. BMI was calculated as body weight/height (kg/m^2^). Waist circumference and hip circumference were measured in cm with a non-stretchable measuring tape in the upright standing position. Hip circumference was measured from the maximum diameter of the hips, and waist circumference was measured with a non-flexible tape measure between the lowest rib and the iliac process and at the narrowest part of the waist above the navel. Thus, waist/height (cm) and waist/hip (cm) ratios were obtained.

### 2.2. PRS Calculation

**Figure 1:**
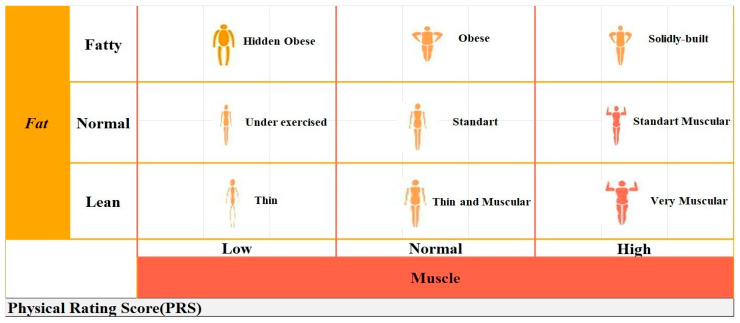
Body types and scoring according to the physical rating obtained from BIA.

The PR derived from BIA categorizes individuals into nine distinct body types based on the ratios of fat and muscle mass [13]. These classifications are as follows:

1. Hidden obese (characterized by low muscle mass and a high fat ratio)
2. Obese (normal muscle mass with a high fat ratio)
3. Solidly built (high muscle mass accompanied by a high fat ratio)
4. Under-exercised (low muscle mass with a normal fat ratio)
5. Standard (normal ratios of both muscle and fat)
6. Standard muscular (high muscle mass and a normal fat ratio)
7. Thin (low ratios of both muscle and fat)
8. Thin and muscular (normal muscle mass with a low fat ratio)
9. Very muscular (high muscle mass and a low fat ratio) (Figure 1).

In our study, 4 body types were obtained in both groups according to PR with Tanita MC-780MA. These are standard, very muscular, hidden obese and obese. The Tanita analyzer automatically calculated the PRS out of 100 points for each body type. For a better understanding of this scoring system, we give an example of a figure showing body types and the score scale (Figure 1).

### 2.3. Blood Sampling

After 8–10 h of fasting, blood was collected in anticoagulant-free gel tubes and centrifuged at 2000× *g* for 10 min after 30 min of clotting, after which the fasting glucose concentration was measured. Glycosylated hemoglobin (HbA1c) was analyzed from whole-blood samples collected in K2EDTA tubes and measured by high-performance liquid chromatography (Premier Hb9210; TrinityBiotech, Co., Wicklow, Ireland).

### 2.4. Statistics

All the statistical analyses were performed using Statistical Package for Social Sciences version 28.0 software for Windows (IBM SPSS Statistics for Windows, Version 28.0. IBM Corp., Armonk, NY, USA).

The assumption of normality for quantitative variables was tested by the Kolmogorov–Smirnov and Shapiro–Wilk tests. Descriptive statistics of the variables are given as the mean ± standard deviation, median (min–max), and n (%) (Table 1). The normality of continuous variables was assessed using the Kolmogorov–Smirnov and Shapiro–Wilk tests. As some variables did not follow a normal distribution, Spearman’s rank correlation coefficient was used to assess the relationships between metabolic age and other parameters (Table 2).

Linear regression analysis was performed to identify independent predictors of metabolic age. A stepwise variable selection method was applied to construct the final regression model; only variables that remained significant after the selection procedure were included in the final models reported in Table 3 and Table 4.

Group comparisons were conducted using the independent samples *t*-test or the Mann–Whitney U test, as appropriate. The Kruskal–Wallis test was used for comparisons involving more than two groups. When a statistically significant difference was detected, pairwise comparisons were performed using the Mann–Whitney U test. To control type I error due to multiple comparisons, Bonferroni correction was applied, and a corrected significance threshold of *p* < 0.0083 was considered.

Sample size was calculated by performing an a priori power analysis. In the power analysis, effect size: 0.3, α error probability: 0.05, power (1 − β error probability): 0.90 were taken. The minimum sample size needed was calculated. One hundred and twenty-two subjects, which is approximately 10% more than the calculated sample size (N = 109), were used. Power analysis was performed using the G*Power (3.1.9.7) package program.

## 3. Results

A total of 122 patients participated in the study. Of the participants, 50 (41.0%) were male and 72 (59.0%) were female. Descriptive statistics and group comparisons of the patients are described in Table 1. According to these results, there was no difference between the sexes in terms of chronological age or met-age (*p* > 0.05). However, the difference in age between the met-age and chronological age was significantly greater in the female group than in the male group (*p* < 0.001).

BMI, degree of obesity and fat mass were significantly greater in females (*p* < 0.001), waist/hip ratios were lower (*p* < 0.001), and waist/height ratios were greater (*p* = 0.001). PRS, muscle mass and lean mass were greater in males than in the females (*p* < 0.001). The difference between sexes in terms of HbA1c and fasting glucose values was not statistically significant (*p* > 0.05).

Correlations between met-age and other variables in both sexes are given in Table 2. According to these results, met-age was positively correlated with chronological age (r = 0.887, *p* < 0.001), BMI (r = 0.370, *p* < 0.001), fat mass (r = 0.368, *p* = 0.001), waist/hip ratio (r = 0.298, *p* = 0.011) and waist/height ratio (r = 0.435, *p* < 0.001) in the females.

In the males, the met-age was positively correlated with chronological age (r = 0.913 **, *p* < 0.001), BMI (r = 0.389 **, *p* < 0.001), degree of obesity (r = 0.316 *, *p* = 0.025), fat mass (r = 0.346 *, *p* = 0.014) and waist/height ratio (r = 0.436 **, *p* = 0.002).

There was a negative correlation between met-age and the PRS in both sexes (male: r= −0.361 *, *p* = 0.001; female: r = −0.307 *, *p* = 0.009).

The coefficients of the multiple linear regression analysis performed to determine the factors affecting met-age are given in Table 3 and Table 4 for both sexes. According to these results, met-age in females is influenced by chronological age, the PRS and the waist-to-height ratio. Accordingly, as chronological age and the waist/height ratio increase and the PRS decreases, met-age increases. R^2^ = 0.983 was found for the linear regression model (Table 3).

The factors affecting met-age in males were chronological age, PRS, degree of obesity and BMI. The linear regression model yielded R^2^ = 0.974. Accordingly, as chronological age, the degree of obesity and BMI increase, and the PRS decreases, the met-age increases (Table 4).

The PRS values corresponding to different body types in the female T2DM group are summarized in Table 5. A statistically significant difference was observed between the body types in terms of PRS values, as evidenced by the Kruskal–Wallis test (*p* < 0.001). Subsequent pairwise comparisons using the Mann–Whitney U test revealed that the standard body type exhibited the highest PRS values, while the obese group demonstrated the lowest. All intergroup comparisons were statistically significant (*p* < 0.001 for all pairwise comparisons), indicating a consistent and robust association between body type and PRS within this cohort.

Table 6 presents the PRS values across different body types in the male T2DM group. The Kruskal–Wallis test indicated a statistically significant overall difference in PRS values among the groups (*p* < 0.001). Pairwise comparisons using the Mann–Whitney U test demonstrated that the standard body type had significantly higher PRS values compared to all other groups (*p* < 0.001 for all comparisons). Similarly, the very muscular group showed significantly higher PRS values than both the hidden obese and obese groups (*p* < 0.001 for both). However, the difference in PRS values between the hidden obese and obese groups was not statistically significant (*p* = 0.147).

## 4. Discussion

This is the first study to examine met-age in T2DM patients with BIA. In both sexes, chronological age and PRS emerged as common determinants significantly influencing met-age (*p* < 0.001). Met-age increases with advancing chronological age and decreasing PRS. Additionally, sex-specific factors were identified: in females, the waist-to-height ratio played a crucial role in the progression of met-age, whereas in males, BMI and obesity grade were found to be significant contributors to its advancement (Table 3 and Table 4).

Previous studies have also underscored the clinical significance of met-age as a biomarker. Rodelo et al. reported a notably elevated met-age in individuals with CVD and found that it increased with chronological age [11]. Additional research supports the diagnostic value of met-age in various metabolic conditions. Alverez et al. showed that met-age was significantly greater than chronological age in patients with metabolic syndrome compared to controls [10]. Similarly, Mehrdad et al. observed that met-age was elevated in both male and female patients with metabolic syndrome relative to controls, proposing it as a potential predictive biomarker [13]. In T2DM, significant changes occur in body composition with advancing chronological age. These changes are characterized by an increase in fat mass accompanied by a decrease in lean body mass and muscle mass [14]. Studies on middle-aged obese individuals with T2DM have shown more visceral fat accumulation and hepatosteatosis as well as decreased muscle mass compared with non-diabetic controls [15].

Although there was no difference between the met-age and chronological age levels of the groups in our study, the met-age–chronological age difference was significantly higher in the female group (Table 1). This may have been due to the lower PRS and waist/hip ratio and higher BMI, waist/height ratio and fat mass in the female T2DM group (Table 1). In terms of sex, although T2DM was diagnosed at an earlier age in male patients, they were reported to have a lower BMI than female patients [16,17]. Compared with male patients, female patients with T2DM were reported to have higher rates of important risk factors, such as overweight, excess fat mass, obesity indices, BMI, and high blood pressure [18,19]. In addition, with advancing chronological age, female T2DM patients have been reported to lose more bone and muscle mass than males in the same age group [20,21]. The correlation between waist-to-height ratio and met-age was statistically significant in both sexes; however, this result was stronger in females (female; *p* < 0.001, male; *p* = 0.002) (Table 2). Furthermore, linear regression analysis confirmed its significant predictive effect on met-age (*p* = 0.024) (Table 3). These results also elucidate why waist-to-height ratio specifically influences met-age in females. Moreover, the waist-to-height ratio is well-recognized as a robust indicator of abdominal and visceral adiposity [22]. In a large-scale study by Qiwei et al., the waist/height ratio was shown to be greater in female T2DM patients aged 18–59 years than in male patients and to be strongly associated with the disease, suggesting that it has a significant effect on the risk of T2DM in the general population [23]. Rodelo et al. found a more significant association of met-age with waist/height ratio than chronological age in individuals with CVD [11]. L. Radzevičienė et al. showed that waist circumference, BMI and high waist/height ratio are important risk factors in female patients with T2DM, compared to controls [24]. In the present study, linear regression analysis revealed that, following chronological age and PRS, BMI and obesity grade were strongly associated with met-age in the male T2DM group (Table 4). It has been shown that BMI is a better predictor of T2DM in male patients with T2DM than in females [25].

The degree of obesity obtained by BIA is a different scale from the World Health Organization’s classification of obesity based on BMI. In a multicentre study conducted by R. Amani, it was pointed out that there were significant differences between BMI and BIA in thin females. Thin females had low BMI but high fat mass. The percentage of fat mass in the body is an important factor when determining the degree of obesity by electrical impedance [26].

No correlation of met-age with fasting glucose and HbA1C reflecting metabolic dysfunction was detected in either group (*p* > 0.05, Table 2). It has been shown that the main determinants of met-age are chronological age and PRS rather than impaired glycemic index. In addition, waist-to-height ratio affected women and BMI/obesity degree affected men. Due to the limited number of patients, we did not investigate the relationship between met-age and PRS with DM complications, but we are planning prospective studies.

In our study, PRS values were found to be highest in individuals with a standard body type, whereas they were significantly lower in those with obese and hidden obese body types (*p* < 0.05) (Table 5 and Table 6). These results suggest that as the proportion of body fat mass increases, PRS decreases. To our knowledge, this is the first study to demonstrate the association between PRS and body types, albeit with a limited sample size. Previous research utilizing BIA has identified a significant relationship between increased body fat mass and a heightened risk of T2DM [27,28]. Females with T2DM have been shown to possess higher body fat mass compared to non-diabetic controls, with this fat accumulation potentially linked to genetic predisposition [29]. The rising prevalence of obesity is strongly correlated with an increased incidence of T2DM and serves as a robust predictor for the onset of the disease [30]. In our study, PRS measurements were obtained using the Tanita MC-780MA device, marking the first use of this scale in the literature. Consequently, we were unable to reference prior studies for comparison. We anticipate that our research will serve as a significant reference for future studies employing similar methodologies. However, due to the limited sample size, we were unable to comprehensively evaluate the relationship between met-age and the four distinct body types. We recommend conducting further investigations with larger cohorts to explore these associations in greater depth and enhance the understanding of this subject.

The preprint of our study was published in researchsquare.com (https://doi.org/10.21203/rs.3.rs-5057249/v1) [31]. In this article, major revisions were made to the title and main text, and a figure defining PRS was created.

## 5. Conclusions

Our study revealed that among female patients, the waist-to-height ratio significantly influenced and contributed to an increase in met-age, whereas in male patients, BMI and obesity grade were identified as the factors driving a notable elevation in met-age.

Chronological age and PRS significantly affected both sexes (*p* < 0.001). Chronological age is a risk factor that cannot be changed, but improvements in PRS, waist/height ratio, BMI and degree of obesity can be achieved so that met-age is younger than chronological age and good metabolic health can be achieved. These results emphasize the importance of understanding the interaction between met-age, anthropometric measurements and diabetes management in different genders.

We considered four different body types in our study and found that PRS was highest in the standard body type and lowest in obese groups, but we could not evaluate the relationship between body types and met-age. Therefore, we recommend an investigation into the relationship between the nine different body types and met-age in T2DM, the determination of the ideal body type and the predictive value of body types on met-age with larger amount of participants in the future.

## Figures and Tables

**Table 1:** Demographic variables, BIA measures and laboratory parameters of the groups.

Variables/Sexes	Total, n = 122	Female, n = 72	Male, n = 50	*p*
Chronological age	56.33 ± 11.36 57.0 (19–85)	56.14 ± 12.41 57.0 (19–85)	56.6 ± 9.77 57.0 (36–85)	0.827 ^&^
Met-age	58.30 ± 12.16 58.0 (19–89)	59.92 ± 12.90 60.0 (19–83)	55.98 ± 10.71 55.0 (39–89)	0.079 ^&^
Met-age–chronological age difference	4.92 ± 3.65 4.0 (0.0–12.0)	6.0 ± 3.79 6.0 (0–12)	3.38 ± 2.80 3.0 (0.0–12.0)	<0.001 *
PRS	62.73 ± 24.63 49.0 (34.0–96.0)	54.76 ± 23.04 45.0 (34–96)	74.20 ± 22.40 91.0 (34–96)	<0.001 *
BMI	30.28 ± 5.20 29.75 (20.30–45.80)	31.96 ± 5.17 31.20 (22.0–45.80)	27.87 ± 4.24 28.0 (20.30–43.6)	<0.001 ^&^
Degree of obesity	23.58 ± 18.20 18.30 (0.94–93.31)	28.56 ± 20.03 26.12 (1.14–93.31)	16.42 ± 12.16 13.04 (0.94–71.06)	<0.001 *
Fat mass	26.02 ± 9.75 24.45 (7.60–51.80)	30.34 ± 8.64 29.55 (14.30–48.0)	19.80 ± 7.74 18.90 (7.60–51.80)	<0.001 ^&^
Muscle mass	54.20 ± 8.48 52.80 (36.60–77.80)	49.95 ± 5.92 50.45 (36.60–62.70)	60.31 ± 7.89 60.9 (40.8–77.8)	<0.001 ^&^
Lean mass	57.12 ± 8.98 55.60 (38.60–81.80)	52.61 ± 6.22 53.15 (38.6–66.0)	63.61 ± 8.38 64.1 (43–81.8)	<0.001 ^&^
Waist/hip ratio	0.89 ± 0.05 0.90 (0.71–1.0)	0.87 ± 0.05 0.87 (0.76–1.0)	0.91 ± 0.05 0.92 (0.71–1.0)	<0.001 *
Waist/height ratio	0.58 ± 0.07 0.57 (0.42–0.83)	0.60 ± 0.08 0.59 (0.45–0.78)	0.55 ± 0.06 0.55 (0.42–0.83)	0.001 ^&^
Fasting glucose	180.20 ± 70.20 170.50 (97.0–395.0)	180.48 ± 68.64 176.0 (98.0–395.0)	179.80 ± 73.10 153.50 (97.0–358.0)	0.958 *
HbA1c	8.39 ± 2.12 7.85 (5.70–14.70)	8.35 ± 2.10 8.20 (5.70–14.70)	8.44 ± 2.17 7.60 (5.80–14.40)	0.817 ^&^

^&^: Independent *t* test, *: Mann–Whitney U, Met-age: metabolic age, PRS: physical rating score, BMI: body mass index, HbA1C: glycosylated hemoglobin.

**Table 2:** Correlations between metabolic age and other variables across sexes.

Variables/Sexes	Female		Male	
	r	*p*	r	*p*
Chronological age	0.887 **	<0.001	0.913 **	<0.001
PRS	−0.307 **	0.009	−0.361 *	0.001
BMI	0.370 **	<0.001	0.389 **	<0.001
Degree of obesity	0.088	0.461	0.316 *	0.025
Fat mass	0.368 **	0.001	0.346 *	0.014
Muscle mass	−0.051	0.670	0.057	0.697
Lean mass	−0.051	0.672	0.041	0.779
Waist/hip ratio	0.298 *	0.011	0.233	0.103
Waist/height ratio	0.435 **	<0.001	0.436 **	0.002
Fasting glucose	−0.023	0.847	0.035	0.809
HbA1c	0.059	0.620	0.069	0.634

* *p* < 0.05; ** *p* < 0.001 (Spearman correlation coefficient), PRS: physical rating score, BMI: body mass index, HbA1C: glycosylated hemoglobin.

**Table 3:** Linear regression results of factors affecting metabolic age in female T2DM group.

Variables	B	95% Confidence Interval for B		*p*
		**Low**	**Upper**	
Chronological age	0.923	0.861	0.985	<0.001
PRS	−0.082	−0.112	−0.053	<0.001
BMI	0.387	−0.031	0.804	0.069
Degree of obesity	−0.032	−0.132	0.069	0.529
Fat mass	−0.017	−0.227	0.193	0.870
Waist/height ratio	39.400	5.280	73.519	0.024
Waist/hip ratio	−4.591	−20.987	11.804	0.578

PRS: physical rating score, BMI: body mass index.

**Table 4:** Linear regression results of factors affecting metabolic age in male T2DM group.

Variables	B	95% Confidence Interval for B		*p*
		**Low**	Upper	
Chronological age	0.990	0.928	1.053	<0.001
PRS	−0.105	−0.136	−0.074	<0.001
BMI	0.623	0.185	1.061	0.006
Degree of obesity	0.118	0.022	0.214	0.017
Fat mass	−0.041	−0.296	0.213	0.744
Waist/height ratio	−23.996	−61.224	13.232	0.200
Waist/hip ratio	7.459	−13.519	28.437	0.477

PRS: physical rating score, BMI: body mass index.

**Table 5:** Relationship between body types and PRS in female T2DM group.

Variables	Standard (n: 17)	Very Muscular (n: 5)	Hidden Obese (n: 25)	Obese (n: 25)	*p*
PRS	92.76 ± 1.67 93.00 (91–96)	69.60 ± 3.13 71.00 (64–71)	44.80 ± 1.00 45.00 (44–49)	35.92 ± 4.94 34.00 (34–49)	<0.001 *

*: Kruskal–Wallis tests, (p^S-V^, p^S-H^, p^S-O^, p^V-H^, p^V-O^, p^H-O^): Pairwise comparisons were performed using the Mann–Whitney U test. To reduce the risk of type I error due to multiple comparisons, Bonferroni correction was applied. PRS: physical rating score.

**Table 6:** Relationship between body types and PRS in male T2DM group.

Variables	Standard (n: 26)	Very Muscular (n: 11)	Hidden Obese (n: 9)	Obese (n: 4)	*p*
PRS	93.65 ± 1.85 93.00 (91–96)	65.64 ± 7.44 67.00 (45–71)	44.56 ± 0.52 45.00 (44–45)	38.00 ± 7.34 34.50 (34–49)	<0.001 *

*: Kruskal–Wallis tests, (p^S-V^, p^S-H^, p^S-O^, p^V-H^, p^V-O^, p^H-O^): Pairwise comparisons were performed using the Mann–Whitney U test. To reduce the risk of type I error due to multiple comparisons, Bonferroni correction was applied. PRS: physical rating score.

## Data Availability

All data generated or analyzed during this study are included in this published article.
